# Does Psychological State Influence the Physiological Response to Cardiac Rehabilitation in Older Adults?

**DOI:** 10.3390/medicina60030361

**Published:** 2024-02-21

**Authors:** Karolina Kowalewska, Kamil Radecki, Błażej Cieślik

**Affiliations:** 1 Institute of Physical Culture Sciences, Jan Dlugosz University, 42-200 Czestochowa, Poland; kowalewskamaja@wp.pl (K.K.); kamil_radecki@onet.pl (K.R.); 2 Healthcare Innovation Technology Lab, IRCCS San Camillo Hospital, 30126 Venice, Italy

**Keywords:** cardiac patients, risk factors, psychological impact, physiotherapy

## Abstract

*Background and Objectives*: Cardiovascular diseases (CVDs) are a major global cause of death. Effective secondary prevention is crucial, involving risk factor modification and cardiac rehabilitation. However, mental factors, particularly depression, exert a significant influence on CVD outcomes by increasing cardiovascular risk and impeding treatment adherence. Therefore, the aim of this study is to assess the impact of psychological state on the effectiveness of rehabilitation in cardiac patients. *Materials and Methods*: Thirty-three patients referred for cardiac rehabilitation participated in a 3-week program, retrospectively categorized into two groups: those with and without depressive symptoms. The functional status of the patients was assessed using the R.A.M.P. protocol exercise test, conducted on a treadmill, during which resting and exercise heart rate (HR), systolic (SBP) and diastolic (DBP) blood pressure, and metabolic equivalent of task (MET) measurements were taken. The Hospital Anxiety and Depression Scale (HADS) and the Perceived Stress Scale (PSS-10) were utilized to evaluate the patients’ psychological state. Stepwise regression explored the psychological factors explaining physiological parameter variance. *Results*: Participants without depressive symptoms exhibited significantly greater improvements in exercise HR (15.58 vs. 1.07; *p* = 0.02), exercise SBP (7.93 vs. −2.05; *p* = 0.05), and exercise METs (1.52 vs. 0.50; *p* = 0.006) compared to those with depressive symptoms. The following predictors were found to be significant: for exercise HR—HADS-D (*r*^2^ = 12%; *p* = 0.04); for exercise DBP—PSS-10 (*r*^2^ = 27%; *p* = 0.002); and for METs—HADS-D and age (*r*^2^ = 26%; *p* = 0.01). *Conclusions*: In conclusion, cardiac rehabilitation improved psychological and physiological parameters in both groups, with greater effectiveness seen in those without depression. Depressive symptoms predicted exercise HR, SBP, and METs, highlighting their role in worsening cardiac disease. Emphasizing psychological factors, including depression and stress, in cardiac rehabilitation can enhance effectiveness and patient outcomes.

## 1. Introduction

Cardiovascular diseases (CVDs) stand as the foremost cause of both global mortality and morbidity. According to the World Health Organization (WHO), in 2015, CVDs resulted in more than 17.7 million deaths, representing 31% of total global fatalities [1]. Considering that numerous risk factors for cardiovascular diseases can be altered (including smoking, physical inactivity, hypertension, unhealthy dietary habits, and disorders related to carbohydrate and lipid metabolism, as well as excess weight and obesity), prevention assumes a pivotal role. This includes the identification, evaluation, and implementation of measures aimed at either eliminating or modifying the identified risk factors [2]. Therefore, cardiac rehabilitation is an important tool in the secondary prevention of cardiovascular diseases [3]. According to the World Health Organization (WHO), cardiac rehabilitation is a set of activities that lead to ensuring the best possible mental, social, and physical conditions for a person with heart disease, so that they can, with their own participation, return to normal professional and family life [4]. Thus, cardiac rehabilitation can be considered a comprehensive and multidirectional approach.

To date, a range of mental disorders and psychosocial factors have been identified that are associated with the development and progression of atherosclerotic disease in the cardiovascular system. These factors include depression and anxiety disorders, certain personality traits, and chronic stress stimuli such as low socio-economic status, lack of social support, and stressful situations at work and in personal life [5]. One of the most frequently occurring and well-documented factors is depressive disorder, which affects one in five adults and is the second most common cause of disability in the USA [6]. Depression is also a major cause of reduced quality of life in patients with cardiovascular diseases and is considered an independent risk factor for serious and adverse cardiovascular events [7]. Estimates conducted in various countries show that reducing the level of risk factors in the population is more effective in lowering the mortality rate from cardiovascular causes than improving therapy for and diagnosis of diseases [8]. Recognized methods that help reduce morbidity, the frequency of rehospitalization, and mortality due to cardiac reasons include both primary and secondary prevention of cardiovascular diseases, understood as lifestyle changes combined with regular physical training and appropriate pharmacotherapy [3].

The interplay between mental state and somatic conditions can manifest in several ways: the impact of mental state on the occurrence and course of somatic disease, the presence of functional disorders, psychological reactions to somatic illness, responses to psychosocial situations, and the co-occurrence of somatic disease and mental disorders [9]. In some somatic diseases, the clinical picture can be significantly altered due to coexisting depressive or anxiety disorders. Excessive sympathetic nervous system activation or reduced parasympathetic activity leads to rhythm disturbances in cells [10]. Patients suffering from post-traumatic stress disorder have an increased risk of somatic diseases, primarily diabetes and cardiovascular diseases [11]. Mental factors significantly affect the course of somatic diseases such as coronary artery disease, peptic ulcer disease of the duodenum and stomach, and atopic dermatitis [12]. Moreover, Haug et al. (2004) have shown a statistically significant relationship between depression, anxiety symptoms, and functional disorders [13]. People suffering from both depressive and anxiety disorders showed the most functional disorders.

In patients with cardiovascular diseases, depression is associated with poor prognosis. The presence of depression after a myocardial infarction independently correlates with a 2- to 4-fold increased risk of subsequent cardiovascular events. This risk is directly proportional to the severity of depressive disorders. Patients with symptoms of depression that are resistant to treatment also have a higher risk of subsequent cardiovascular events [14]. Additionally, individuals after a myocardial infarction are three times more likely to experience depressive disorders than the general population [15]. In depression, apart from a low mood and other symptoms affecting the mental state, its somatic symptoms are also characteristic. These symptoms include sleep and appetite disorders; fatigue; indigestion; nausea; dizziness and headaches; frequent pain in muscles, joints, the abdominal cavity, and chest; as well as heart palpitations [16,17]. Additionally, it is believed that depressive disorders can influence the development of heart diseases, hypertension, diabetes, or stroke [18]. Depressive disorders significantly disrupt the optimal treatment of patients with CVDs, mainly through worsening adherence to a healthy lifestyle and reducing the effectiveness of therapies recommended by specialists.

The primary aim of this study is to evaluate and confirm the potential influence of mental state on the effectiveness of cardiac rehabilitation. By gaining a deeper understanding of the two-way relationship between cardiovascular disease and psychological well-being, particularly in terms of disparities in rehabilitation outcomes among patients with symptoms of depression, we seek to address whether an increase in physical activity levels can lead to a reduction in depression indicators in cardiac patients.

## 2. Materials and Methods

### 2.1. Study Design and Participants

This study was structured as a retrospective cohort study with a post hoc subgroup analysis. The design and methodology of the study were a priori reviewed and subsequently approved by the Institutional Review Board (IRB) at Jan Długosz University in Częstochowa (Poland), under the approval number KE-U/20/2022. Prior to participation, written informed consent was obtained from each participant.

The research was conducted at the Provincial Specialist Hospital of the Blessed Virgin Mary in Częstochowa, Poland, specifically within the cardiology department. Initially, 33 individuals, both women and men, were selected for the study based on inclusion criteria. This study focused on patients in Phase II of cardiac rehabilitation, aged 60 years and over, diagnosed with cardiovascular diseases including atherosclerosis, hypertension, coronary heart disease, ischemic heart disease, peripheral artery disease, rheumatic heart disease, deep vein thrombosis, pulmonary embolism, and post-myocardial infarction. The exclusion criteria included psychotic symptoms, bipolar disorder, or other serious psychiatric disorders; initiation of psychiatric treatment or individual psychological therapy during the research project; use of antidepressant medications; cognitive impairments preventing questionnaire completion; consciousness disorders; and patient refusal. Participants were retrospectively categorized into two groups based on their scores on the depression subscale of the Hospital Anxiety and Depression Scale (HADS): those exhibiting symptoms of depression (scores above 7) and those without depressive symptoms (scores between 0 and 7). Before starting rehabilitation, all participants completed questionnaires and underwent endurance tests. Each patient actively participated in the rehabilitation and, after its completion, repeated the endurance test and filled out one of the questionnaires again.

### 2.2. Cardiac Rehabilitation

The cardiac rehabilitation program spanned 3 weeks, featuring a variety of group exercises and activities. The daily routine included morning ‘start-up’ exercises, afternoon sessions, interval training on bicycles, and relaxation to music. Morning sessions, lasting 20 min, involved standing cardiovascular exercises accompanied by music. Afternoon exercises, which were 45 min long, were conducted with the patients seated on chairs. These sessions were divided into three segments: the first focused on cardiovascular exercises, the second incorporated equipment like balls or sticks, and the third emphasized breathing exercises. Patients’ pulse rates were measured before and after these sessions, along with blood pressure post session. Following this, a 20 min interval training on bicycles was conducted, with individualized intensity levels; blood pressure was again measured before and after this activity. Post lunch, the program included relaxation sessions with music, where patients unwound in a semi-reclining position on loungers. The exercise regimen was scheduled from Monday to Friday, with only afternoon exercises on Saturdays and a rest day on Sundays. For patients unable to join group activities, individual bedside sessions were offered in their rooms. Additionally, throughout the rehabilitation process, patients had access to a psychologist for individual consultations.

### 2.3. Outcome Measures

The assessment of the psychological and physiological status of patients was conducted at the baseline (week 0) and after the completion of the cardiac rehabilitation cycle (week 3).

For assessing psychological states, we utilized the Polish version of the Hospital Anxiety and Depression Scale (HADS) and the Perceived Stress Scale (PSS-10). The HADS is a 14-item instrument, comprising two subscales: one for anxiety (HADS-A) with 7 items, and the other for depression (HADS-D), also with 7 items. Participants were asked to rate each item based on their experiences over the past week, choosing from four response options that best described their feelings. The scores on each subscale range from 0 to 21, with higher scores indicating more severe symptoms of anxiety or depression. A cutoff score of 0–7 points on each subscale is considered within the normal range [19]. The PSS-10 is employed to gauge a patient’s perceived stress. This tool comprises 10 questions focused on various subjective feelings associated with personal issues, life events, behaviors, and coping strategies. The scoring on the PSS-10 is structured such that higher scores indicate elevated levels of perceived stress, as referenced in the literature [20]. To accurately capture stress levels prior to the intervention, the PSS-10 assessment was conducted solely at baseline.

Physiological parameters were assessed under both resting and exercise conditions. The exercise conditions were achieved through the R.A.M.P. protocol exercise test, conducted on a treadmill. This test dynamically adjusts the load based on each patient’s capabilities, with individually tailored loads and an exercise duration of approximately 10 min. Throughout the test, patients were connected to an EKG, providing a detailed view of heart function during exercise [21]. We analyzed heart rate (HR in beats per minute, bpm), systolic blood pressure (SBP), and diastolic blood pressure (DBP), with measurements recorded in millimeters of mercury (mmHg) for SBP and DBP. To evaluate participants’ exercise capacity and endurance levels, we calculated metabolic equivalents (METs) by determining the ratio of the working metabolic rate to the resting metabolic rate during physical activities, providing a comprehensive measure of energy expenditure [22]. In addition, to monitor the perceived intensity of the exercises in our study, we recorded participants’ Ratings of Perceived Exertion (RPEs) at regular two-minute intervals during the exercise sessions using the Borg Scale, which has a range from 6 to 20. This scale is a widely recognized method for participants to subjectively assess and report their perceived effort during physical activities [23].

### 2.4. Data Analysis

Data analysis was conducted using JASP software (v0.16.3). Descriptive statistics for categorical variables were presented as frequencies and percentages. For continuous variables, the mean and standard deviation (SD) were provided. The Shapiro–Wilk test showed a normal distribution of quantitative data. Demographic variables at the beginning of the study were compared between groups using the t-test for independent samples (continuous variables) and the χ^2^ test (categorical variables). The *t*-test for dependent samples was used to analyze intervention effects (before vs. after intervention). ANOVA was used to compare groups before and after the intervention. For ANOVA, partial eta squared (*η*p^2^) was calculated, with values above 0.13 indicating a large effect, values between 0.6 and 0.13 indicating a medium effect, and values below 0.5 indicating a small effect. Stepwise regression was used to investigate which variables could predict changes in the effectiveness of cardiac rehabilitation. In the model, the change in functional parameter scores (HR, SBP, DBP, SBP, DBP, METs, RPE) was set as the dependent variable, while the initial scores (week 0) of psychological parameters (PSS-10, HADS, HADS-D, HADS-A) were included as potential predictors. Statistical significance was set at *α* < 0.05.

## 3. Results

### 3.1. Participants Characteristics

Of the 54 potential participants available, 33 were assigned to the study based on the inclusion criteria. The average age of the participants was 72.15 years (SD 7.70). The study comprised 22 female and 11 male participants. The diagnostic breakdown among the participants was as follows: 10 (30.3%) had STEMI, 8 (24.2%) had NSTEMI, and 15 (45.5%) had HF. All participants who met the qualification criteria successfully completed the study. As indicated in Table 1, the statistical analysis revealed no significant differences in the prevalence of these conditions, or in baseline exercise capacity and stress levels, between the group with symptoms of depression and the group without such symptoms.

### 3.2. Effectiveness of the Intervention

Among the physiological parameters, notable changes were observed in the exercise HR of the non-depressive group, which exhibited a significant increase post intervention, rising from 88.16 (SD 20.85) to 103.74 (SD 21.14), representing an increase of approximately 17.7% (*p* < 0.001). Furthermore, METs showed significant improvement in both groups. The non-depressive group experienced a more pronounced increase, from 3.68 (SD 1.69) to 5.21 (SD 1.81), which translates to a 41.6% improvement (*p* < 0.001) (Table 2).

In terms of psychological well-being, assessed using the HADS, significant improvements were noted, particularly in the group with depressive symptoms. The HADS-D score in this group decreased notably from 11.21 (SD 2.33) to 8.64 (SD 2.10), showing a 22.9% reduction (*p* = 0.002). The overall HADS score, encompassing both anxiety and depression, also showed significant improvements. The depressive symptoms group exhibited a decrease from 20.86 (SD 4.37) to 16.57 (SD 4.03), a reduction of 20.6% (*p* = 0.001). In the non-depressive group, the overall HADS score decreased from 13.37 (SD 4.86) to 9.05 (SD 4.14), translating to a 32.3% improvement (*p* < 0.001) (Table 2).

**Figure 1:**
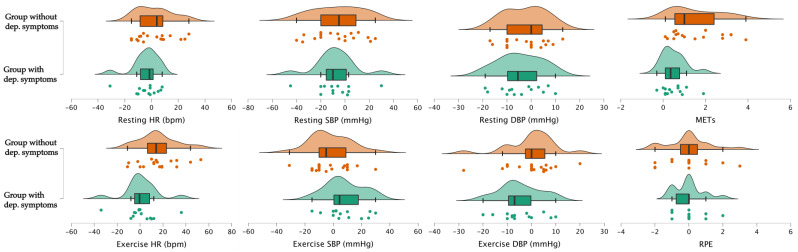
Raincloud plots illustrating pre–post differences in physiological parameters. HR: heart rate; bpm: beats per minute; SBP: systolic blood pressure; DBP: diastolic blood pressure; METs: metabolic equivalents; RPE: ratings of perceived exertion.

Table 3 and Figure 1 both relate to pre–post differences in physiological parameters; the table presents the mean differences in changes, while the figure displays these differences through raincloud plots for each group. The data analysis revealed notable differences in the changes in physiological parameters between participants with and without depressive symptoms. A significant difference was observed in the exercise HR, where participants without depressive symptoms exhibited a notable average increase of 15.58 (SD 16.77), compared to an increase of 1.07 (SD 15.04) in those with depressive symptoms (*p* = 0.02). Furthermore, there was a statistically significant difference in the exercise SBP, with an average increase of 7.93 (SD 13.29) in the depressive symptoms group, in contrast to a slight average decrease of −2.05 (SD 14.16) in the non-depressive group (*p* = 0.05). Additionally, the change in METs was significantly different between the groups: the non-depressive group showed an average increase of 1.52 (SD 1.18), whereas the depressive group showed a lesser increase of 0.50 (SD 0.57) (*p* = 0.006).

### 3.3. Correlation and Prediction Analysis

**Figure 2:**
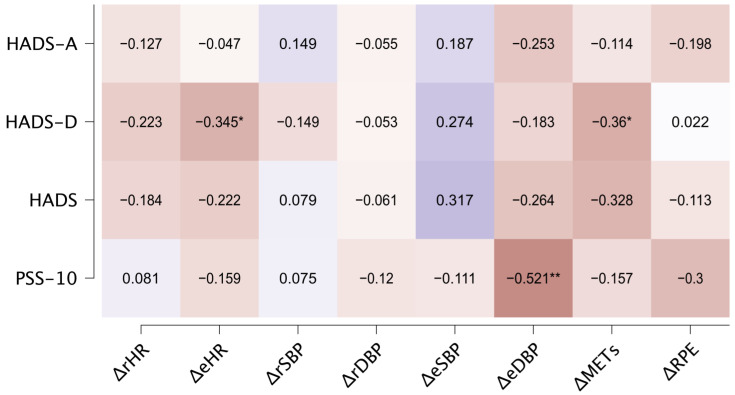
Correlation heatmap of baseline psychological parameters and changes in resting (*r*) and exercise (e) physiological parameters. HR: heart rate; SBP: systolic blood pressure; DBP: diastolic blood pressure; METs: metabolic equivalents; RPE: ratings of perceived exertion.* *p* < 0.05; ** *p* < 0.01.

In assessing the correlations, a statistically significant relationship was found between baseline HADS-D scores and the change in exercise HR (*r* = −0.35; *p* < 0.05). Additionally, a significant correlation was observed between baseline HADS-D and the change in post-intervention METs (*r* = −0.36; *p* < 0.05). Furthermore, baseline PSS-10 scores showed a significant correlation with the change in exercise DBP (*r* = −0.52; *p* < 0.01) (Figure 2).

To determine how much depression, anxiety, and stress can explain the statistically significant amount of variance in physiological parameters, stepwise regression was used. All physiological variables were analyzed, but only baseline HADS-A, HADS-D, PSS-10, and age were considered as predictors in subsequent stages. For the exercise HR parameter, baseline HADS-D emerged as an important predictor, accounting for 12% (*r*^2^ = 0.12) of the variance in these models (*p* = 0.05). A one-point change in baseline HADS-D leads to a decrease in exercise HR by 1.58 beats per minute. Baseline PSS-10 was a significant predictor for exercise DBP, accounting for 27% (*r*^2^ = 0.27) of the variance in the models (*p* = 0.002). A one-point change in baseline PSS-10 leads to a decrease in exercise DBP by 1.29 mmHg. Two significant predictors for change in METs were age and baseline HADS-D, accounting for 26% (*r*^2^ = 0.26) of the variance (*p* = 0.01). A one-year change in age leads to a decrease in METs by 0.05 METs, and a one-point change in baseline HADS-D results in a decrease in METs by 0.10 METs (Table 4).

## 4. Discussion

This study aimed to evaluate and confirm the influence of mental state on the effectiveness of cardiac rehabilitation, focusing on the intervention’s impact on psychological parameters in the group with depression symptoms. The intervention resulted in improvements across all psychological metrics, with reductions of 17%, 23%, and 21% in HADS-A, HADS-D, and overall HADS, respectively. Moreover, we observed improvements in individuals without depression symptoms, with a notable decrease in anxiety disorders following a physical activity intervention. This may suggest that cardiac rehabilitation, understood as physical exertion, reduces depressive and anxious symptoms in patients. Research from various studies consistently demonstrates the positive effects of physical activity on mental health and quality of life across different age groups. Sertel et al.’s study with 3041 individuals showed improvements in life quality in adult, middle-aged, and elderly groups, emphasizing the role of physical activity in alleviating anxiety and depressive symptoms [24]. Complementing these findings, da Silva et al. observed significant reductions in depression and anxiety following a 12-week aquatic training program for individuals aged 50 to 80, particularly in those with pre-existing depression, along with improvements in functional autonomy and oxidative stress [25]. Similarly, Blumenthal et al.’s study on 202 individuals with major depressive disorders revealed that both supervised and home-based aerobic exercises were as effective as antidepressant medication in reducing depressive symptoms [26]. These results align with a literature review by Gieroba, which highlights the importance of physical exercises, including strength and aerobic training, in reducing depression and anxiety disorders, improving cognitive functioning, and enhancing overall well-being, especially in older individuals [27]. From a physiological perspective, individuals with depression symptoms undergoing cardiac rehabilitation exhibited notable improvements, including a 15.20% increase in metabolic equivalent and a 6.22% rise in exercise systolic blood pressure, highlighting the role of physical activity in enhancing endurance and blood pressure regulation. This is corroborated by studies from Lavie et al. [28] and Oeland et al. [29], which demonstrated significant enhancements in endurance, VO_2_max, and other physiological parameters, particularly in older patients and those with heart failure and depressive/anxiety disorders.

The intervention’s impact on physiological parameters, including exercise heart rate, systolic blood pressure, and metabolic equivalent, was more pronounced in the group without depression symptoms, indicating that depressive disorders may reduce the effectiveness of cardiac rehabilitation. This finding is supported by Shen et al.’s research on 142 cardiac rehabilitation patients, which identified that optimism and social support improved post-treatment physical condition, while depressive symptoms required ongoing treatment [30]. Similarly, Turner et al.’s study, linking medical records with lifestyle and hospitalization data of 389 cardiac rehabilitation patients, reinforced the significant influence of depression and anxiety on rehabilitation outcomes [31]. These studies collectively highlight the importance of psychosocial factors in cardiac rehabilitation effectiveness.

Moreover, depressive disorders significantly predicted exercise heart rate, explaining 12% of the variance in physiological parameters. A one-unit increase in HADS-D was associated with a 1.58-beats-per-minute decrease in exercise heart rate, an important cardiovascular and autonomic nervous system activity indicator [32]. Depression, by stimulating the sympathetic system and increasing corticotropin-releasing hormone levels, can lead to increased heart rate, a known risk factor for cardiac events. Supporting this, Pratt et al.’s observational study of 1551 individuals found that major depressive disorders heightened the risk of myocardial infarction [33]. Further, a study by Barefoot and Schroll involving 730 individuals over 27 years revealed that high depressive symptoms significantly increased the risk of myocardial infarction and mortality, underlining the profound impact of depression on heart health [34]. Additionally, stress emerged as a significant predictor for exercise diastolic blood pressure in this study, accounting for 27% of the variance in physiological parameters. Each one-point increase in the PSS-10 stress scale corresponded to a 1.29 mmHg decrease in exercise diastolic blood pressure. Like depressive disorders, stress activates the sympathetic system, influencing blood rheology and immune responses [35]. Stress triggers pro-inflammatory and procoagulant actions, increasing platelet aggregation, plasma clotting factors, and altering blood flow. Both acute and chronic stress can lead to chronic vascular inflammation and accelerated atherosclerosis, a key contributor to myocardial infarction [36]. This was confirmed by Li et al.’s review, which identified chronic stress as an independent risk factor for atherosclerosis development, a primary cause of acute cardiovascular events [37].

Age and depressive symptoms significantly predict the METs in cardiac rehabilitation, explaining 26% of the variance. Each unit increase in depressive symptoms (measured by HADS-D) leads to a 0.10 decrease in METs, highlighting depression’s role in exacerbating cardiac disease morbidity and event occurrence. This finding underscores the impact of depression in exacerbating morbidity and the likelihood of adverse events in cardiac disease. Importantly, a large-scale study involving 7893 individuals established a connection between depression and an elevated risk of ischemic heart disease, as well as increased mortality rates in men [38]. Depression, often characterized by diminished motivation, can negatively influence the effectiveness of cardiac rehabilitation [39]. This is due to the fact that motivation plays a crucial role in determining the intensity of physical activity undertaken during rehabilitation.

### 4.1. Clinical Implications

This study underscores the importance of considering the mental state of patients undergoing cardiac rehabilitation and highlights the potential for cardiac rehabilitation programs to reduce depressive and anxious symptoms. Tailored approaches for individuals with depressive disorders should be integrated into rehabilitation programs to enhance their effectiveness. Feasible tools such as meditation [40], emerging new technology interventions [41,42], or tai chi [43] can provide crucial psychological support for CVD patients. Psychosocial factors, including depression and stress, should be routinely assessed and addressed during rehabilitation, with a focus on promoting optimism and social support. Additionally, depression assessment and management should be a priority, given its impact on exercise heart rate and metabolic equivalent. Age-related differences in motivation should be considered when designing rehabilitation programs, and stress management strategies should be incorporated to improve blood pressure regulation. In summary, a holistic approach that addresses both physiological and psychological aspects is essential for optimizing cardiac rehabilitation outcomes and patient well-being.

### 4.2. Limitations

While this study provides valuable insights into the influence of mental state on cardiac rehabilitation outcomes, it is important to acknowledge several limitations. Firstly, this study’s retrospective cohort design with post hoc subgroup analysis and the lack of a control group may introduce inherent biases and limit the establishment of causality. Secondly, the relatively small sample size of 33 individuals from a single cardiology department in Poland may not be fully representative of broader populations, and the exclusion criteria, including the use of antidepressant medications, cognitive impairments, and consciousness disorders, could lead to selection bias. Thirdly, this study’s focus on patients aged over 54 with specific cardiovascular diseases may limit the generalizability of the findings to a broader range of cardiac rehabilitation patients. Additionally, the three-week duration of the cardiac rehabilitation program may not fully capture long-term outcomes, and longer follow-up periods would be beneficial to assess the sustainability of the observed improvements. Furthermore, the lack of a control group and the absence of randomization in the intervention may affect the ability to draw definitive conclusions about the effectiveness of cardiac rehabilitation in reducing depressive and anxious symptoms. Lastly, this study primarily relied on self-reported scales to assess psychological parameters, and while these are valuable, more objective measurements such as cortisol levels or other physiological markers could provide further insights into the relationships observed.

## 5. Conclusions

In our study, we observed that cardiac rehabilitation contributed to improvements in both psychological and physiological parameters across participants, regardless of their psychological state. This highlights the potential benefits of rehabilitation in mitigating symptoms of depression and anxiety. However, the intervention appeared to yield more pronounced benefits in individuals without depression symptoms, suggesting the possibility that depressive symptoms could hinder the efficacy of rehabilitation. Our analysis also revealed that psychological state could be a significant predictor of exercise-related physiological improvement, especially with an increase in depressive symptoms associated with reduced METs. These observations underscore the need to integrate psychological considerations, especially regarding depression and stress, within cardiac rehabilitation programs to potentially improve their effectiveness and patient outcomes. However, it is important to interpret these results with caution due to this study’s limitations, particularly the small sample size and the absence of a control group. These factors may affect the generalizability of the findings and underscore the necessity for further research with larger, more diverse populations and controlled study designs to validate and expand upon our observations.

## Figures and Tables

**Table 1:** Participants’ characteristics.

Variable	Total	Participants with Depressive Symptoms	Participants without Depressive Symptoms	*p* Value
*n*	33	14	19	
Age, years, mean (SD)	72.15 (7.70)	73.43 (4.55)	71.21 (9.39)	0.42 ^a^
Body mass, kg, mean (SD)	75.09 (13.64)	74.34 (12.62)	75.64 (14.66)	0.79 ^a^
Body height, m, mean (SD)	1.63 (0.10)	1.64 (0.10)	1.62 (0.09)	0.46 ^a^
BMI, kg/cm^2^, mean (SD)	28.46 (5.40)	27.65 (4.53)	29.05 (5.94)	0.19 ^a^
Normal (BMI 18.5–24.9), *n* (%)	9 (27.3)	4 (28.6)	5 (26.3)	0.89 ^b^
Overweight (BMI 25–29.9), *n* (%)	15 (45.4)	6 (42.8)	9 (47.4)	0.80 ^b^
Obese (BMI > 30), *n* (%)	9 (27.3)	4 (28.6)	5 (26.3)	0.18 ^b^
Diagnosis				
STEMI, *n* (%)	10 (30.3)	3 (21.4)	7 (36.8)	0.34 ^b^
NSTEMI, *n* (%)	8 (24.2)	4 (28.6)	4 (21.1)	0.62 ^b^
HF, *n* (%)	15 (45.5)	7 (50.0)	8 (42.1)	0.65 ^b^
Hypertension, *n* (%)	8 (24.2)	3 (21.4)	5 (26.3)	0.75 ^b^
Atherosclerosis, *n* (%)	4 (12.1)	1 (7.1)	3 (15.8)	0.16 ^b^
METs, mean (SD)	3.51 (1.61)	3.29 (1.51)	3.68 (1.69)	0.49 ^a^
PSS-10, mean (SD)	22.73 (3.91)	23.36 (4.34)	22.26 (3.60)	0.93 ^a^

SD: standard deviation; BMI: body mass index; STEMI: ST segment elevation myocardial infarction; NSTEMI: non-ST segment elevation myocardial infarction; HF: heart failure; METs: metabolic equivalents; PSS: Perceived Stress Scale. ^a^ *t*-test; ^b^ Chi-square test.

**Table 2:** Mean values (SD) of physiological and psychological parameters.

Variable	Participants with Depressive Symptoms (*n* = 14)			Participants without Depressive Symptoms (*n* = 19)		
	Baseline	Post Intervention	*p* Value	Baseline	Post Intervention	*p* Value
Physiological parameters						
Resting HR	74.29 (10.90)	70.36 (9.18)	0.16	70.37 (9.59)	72.79 (12.94)	0.44
Exercise HR	86.79 (15.96)	87.86 (16.54)	0.79	88.16 (20.85)	103.74 (21.14)	<0.001
Resting SBP	119.86 (12.73)	111.79 (17.84)	0.09	119.42 (19.25)	113.74 (17.96)	0.24
Resting DBP	70.00 (9.20)	65.50 (7.74)	0.08	72.00 (9.29)	69.53 (8.17)	0.23
Exercise SBP	127.29 (17.55)	135.21 (18.20)	0.04	136.11 (17.80)	134.05 (14.01)	0.54
Exercise DBP	75.29 (7.82)	70.57 (6.79)	0.06	76.26 (9.29)	76.26 (6.70)	1.00
METs	3.29 (1.51)	3.79 (1.78)	0.01	3.68 (1.69)	5.21 (1.81)	<0.001
RPE	13.14 (1.10)	13.14 (1.23)	1.00	13.00 (1.33)	13.00 (0.94)	1.00
Psychological parameters						
HADS-A	9.64 (3.46)	7.93 (3.63)	0.04	7.95 (3.99)	5.84 (3.83)	0.01
HADS-D	11.21 (2.33)	8.64 (2.10)	0.002	4.90 (1.94)	3.74 (2.37)	0.10
HADS	20.86 (4.37)	16.57 (4.03)	0.001	13.37 (4.86)	9.05 (4.14)	<0.001

HR: heart rate; SBP: systolic blood pressure; DBP: diastolic blood pressure; METs: metabolic equivalents; RPE: ratings of perceived exertion; HADS: Hospital Anxiety and Depression Scale (A for Anxiety and D for Depression).

**Table 3:** Mean difference (SD) in changes in physiological parameters between groups.

Variable	Participants with Depressive Symptoms (*n* = 14)	Participants without Depressive Symptoms (*n* = 19)	*p* Value
Resting HR	−3.93 (9.83)	2.42 (13.26)	0.14
Exercise HR	1.07 (15.04)	15.58 (16.77)	0.02
Resting SBP	−8.07 (16.61)	−5.68 (20.36)	0.72
Resting DBP	−4.50 (8.94)	−2.47 (8.64)	0.52
Exercise SBP	7.93 (13.29)	−2.05 (14.16)	0.05
Exercise DBP	−4.71 (8.62)	0.00 (10.15)	0.17
METs	0.50 (0.57)	1.52 (1.18)	0.006
RPE	0.00 (0.88)	0.00 (1.29)	1.0

HR: heart rate; SBP: systolic blood pressure; DBP: diastolic blood pressure; METs: metabolic equivalents; RPE: ratings of perceived exertion.

**Table 4:** Predictors of change in physiological parameters.

Variable	*B*	Beta	*t*	*p* Value	*F*	*R* ^2^
Exercise HR				0.04	4.18	0.12
HADS-D	−1.58	−0.35	−2.04			
Exercise DBP				0.002	11.56	0.27
PSS-10	−1.29	−0.52	−3.40			
METs				0.01	5.20	0.26
Age	−0.05	−0.36	−2.27			
HADS-D	−0.10	−0.36	−2.27			

HR: heart rate; HADS: Hospital Anxiety and Depression Scale (A for Anxiety and D for Depression); DBP: diastolic blood pressure; PSS-10: Perceived Stress Scale; METs: metabolic equivalents.

## Data Availability

Data are available upon reasonable request to the corresponding author.
